# Thermographic Behaviour of the Orbicularis Oris Muscle Under Different Provocative Tests

**DOI:** 10.1111/joor.70117

**Published:** 2025-11-19

**Authors:** Patrícia Vieira Salles, Amanda Freitas Valentim, Maria Luiza Neves Caldeira, Denise Sabbagh Haddad, Renata Maria Moreira Moraes Furlan, Andréa Rodrigues Motta

**Affiliations:** ^1^ Speech‐Language‐Hearing Department at the Pontifical Catholic University of Minas Gerais – PUC Minas Belo Horizonte Minas Gerais Brazil; ^2^ Federal University of Minas Gerais – UFMG Belo Horizonte Minas Gerais Brazil; ^3^ Pontifical Catholic University of Minas Gerais – PUC Minas Belo Horizonte Minas Gerais Brazil; ^4^ Dentistry Department at the University of São Paulo – USP São Paulo São Paulo Brazil; ^5^ Speech‐Language‐Hearing Department at the Federal University of Minas Gerais – UFMG Belo Horizonte Minas Gerais Brazil

**Keywords:** lip, muscle contraction, skeletal muscle, skin temperature, speech, language and hearing sciences, thermography

## Abstract

**Background:**

Infrared thermography is an objective method for investigating muscle functioning, enabling inferences about physiology and therapeutics. This noninvasive and non‐ionising imaging diagnostic method allows real‐time visualisation of the vascular and musculoskeletal systems through skin microcirculation dynamics. It transforms information about the human body's thermal radiation, captured via infrared radiation, into an analysable image.

**Objective:**

To describe the thermographic behaviour of the orbicularis oris muscle during sustained contraction and chewing tasks.

**Methods:**

The study included 56 healthy women aged 18–52 years, who underwent thermographic evaluations before, during, and after performing sustained contraction and chewing tasks. The orbicularis oris muscle was analysed qualitatively and quantitatively using anatomical thermal areas and Student's *t*‐test to compare mean temperature data.

**Results:**

Qualitative analysis revealed temperature changes during the provocative tasks. A comparison of mean temperatures showed a significant temperature increase, corroborating the qualitative findings. The mean temperature variation per task was as follows: 0.57°C during lip compression, 0.20°C during lip protrusion, 0.57°C while chewing peanuts, 0.43°C while chewing crackers, and 0.37°C while chewing a bread roll. There was a decrease in mean temperature during the intervals between tasks, though insufficient to return to baseline levels, indicating a cumulative temperature effect between tasks.

**Conclusion:**

The orbicularis oris muscle temperature increased during the provocative tasks. The 2‐min interval between tasks was insufficient for the resting temperature to return to baseline levels. These findings confirm that thermography is an effective method for identifying such temperature changes.

## Graphical abstract

Study design showing the thermographic region of the orbicularis oris muscle (Picture 1), the foods used for chewing and the increase in mean temperature after the provocative tests (Picture 2).

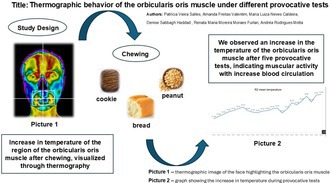

## 1 Introduction

Humans are homeothermic, meaning they can maintain their body temperature regardless of the activity or environment [1, 2]. This temperature regulation can be explained through thermoregulation—that is, the body's integrative physiological responses aimed at keeping the core temperature around 37°C (degrees Celsius) [1]. These responses are primarily coordinated by the hypothalamus [1] and sustained through heat transfer via the body's blood flow [3].

The human body transfers heat primarily through conduction between different body structures, with particular interest in the heat exchange between the facial skeletal muscles and the skin [4]. Heat is transferred by conduction from the region of higher temperature to the region of lower temperature within the body [5]. All body cells produce heat by converting metabolic energy into mechanical and thermal energy. The latter increases significantly during exercise due to dynamic skeletal muscle contraction [4, 6].

The orbicularis oris muscle plays a significant role in various functions of the oral sensorimotor system, such as suction, chewing, swallowing, speech, breathing and facial expression [7, 8]. Thus, it is of particular interest to speech‐language‐hearing pathologists specialising in oral motor therapy, as they aim to understand both the factors that influence it and are influenced by it. This understanding is essential for orofacial function rehabilitation through muscular and functional training. Strategies can be developed to rehabilitate functional impairments—such as in cases of deleterious oral habits, mouth breathing, and facial paralysis—by understanding how orofacial functions behave in healthy muscles [7, 8, 9, 10].

Knowledge about muscle training developed in sports medicine has been incorporated into other health fields, such as rehabilitation [11]. Functional training applies loads to the musculoskeletal system to gain strength and improve functioning. An overload occurs when a function is demanded beyond resting levels. Proper training requires progressive and intermittent overloads [12], observable in thermograms as they show local increases in temperature associated with increased blood flow [6, 13, 14, 15, 16].

Muscle behaviour can be studied in different ways: dynamically and through clinical evaluation [17, 18] and electromyographic assessment [19, 20]. However, none of these methods allow for real‐time visualisation of the vascular and musculoskeletal systems through the dynamics of skin microcirculation—which is possible with infrared thermography, a noninvasive and non‐ionising imaging diagnostic method [21, 22, 23]. Infrared thermography uses infrared radiation to transform the human body's thermal radiation data into analysable images [3, 22, 23]. This objective method investigates muscle function for inferences about physiology and therapeutics, as already used in Medicine and Dentistry [6, 13, 15].

The orbicularis oris muscle can be thermographically analysed through its anatomy, using predefined areas divided into quadrants to facilitate a better understanding of the muscle [24]. It is known that the smaller the area studied, the higher the resolution of the thermographic image [25]. Given the gap in the literature regarding information on temperature distribution in the orbicularis oris muscle and the temperature variation resulting from its activity, it is crucial to study its temperatures in healthy individuals, believing that the activities carried out will increase the temperature. This understanding is fundamental for speech‐language‐hearing pathologists' practice and comprehension of its functional dynamics.

Hence, this study aimed to describe the thermographic behaviour of the orbicularis oris muscle during sustained contraction and chewing tasks.

## 2 Method

This study was approved by the Research Ethics Committee of the Pontifical Catholic University of Minas Gerais (CEP‐PUC Minas) under number 4.972.914—CAAE 50639721.0.0000.5137 and by the Research Ethics Committee of the Federal University of Minas Gerais (COEP‐UFMG) under number 3.695.491—CAAE 21641019.5.0000.5149. The study complied with Resolution no. 466/12 of the Brazilian National Health Council and the principles of the Declaration of Helsinki. All participants signed an informed consent form before being included in the study.

After defining the sample size through sample calculation, the study comprised 56 females, aged 18–52 years, with a mean age of 25.7 years and a standard deviation of 8.04. The calculation showed a 95% statistical power to detect a 0.3°C difference between means before and after the provocative test, considering a 0.78°C standard deviation for the temperature [23]. The study used a 0.05 significance level, Student's paired *t*‐test, and the PASS 11 software (PASS 11. NCSS LLC. Kaysville, Utah, USA. www.ncss.com).

The sample was invited directly to participate, encompassing adult women aged 18–60 years who self‐identified as healthy in the study. The evaluation was scheduled as the invited women agreed to participate in the study according to their and the researcher's availability. Data collection took 1 year and 6 months. It excluded those with a fever on the day of the exam; who did not follow the thermography instructions, according to the criteria of the American Academy of Thermology [25, 26]; who had skin wounds or scars; who were undergoing dental treatment; or who had a history of facial trauma, facial pain, headaches, or symptoms of rhinitis/sinusitis [27]. All participants underwent a medical history survey for identification and general health data.

The data were collected at the Speech‐Language‐Hearing Functional Health Observatory at the Federal University of Minas Gerais. The thermographic images were captured following criteria established by the American Academy of Thermology and confirmed by the TISEM checklist [25, 26] regarding the environment's suitability and the participant's preparation.

Fluorescent lights illuminated the examination, whose humidity was maintained at around 60%, and the temperature was set at 21°C. The room temperature variation did not exceed ±1°C, and the relative humidity did not exceed ±3% [26]. The temperature and humidity values were monitored with a Homis brand thermal hygrometer. These precautions are essential to minimise the environment's influence on image acquisition [25, 28].

The participants remained in the room for 20 min to acclimate their body temperature, with their hair tied up and covered with a cap, without wearing accessories, makeup, or sunscreen, dressed in a strapless top or bra, and barefoot, wearing only a foot cover [26].

They were instructed to prepare for thermography as follows: on the day of the exam, they should not use any products that cover the skin (such as sunscreen or makeup), hair dryers, flat irons, hats, caps, headbands, or bandages; for 2 h before the exam, they should not shower or wash their face with hot water, use the phone, wear headphones, or chew gum; if they felt thirsty, they could drink water at room temperature, but they should not consume coffee 4 h before the exam; in the 24 h preceding the thermography, participants should refrain from physical activity, massage, acupuncture, sauna, photobiomodulation, or ozone therapy, and from consuming alcoholic beverages; they should also temporarily suspend any non‐essential medication [26].

The images were captured with a FLIR A315 thermographic camera, positioned on a tripod at a standardised 1‐m distance from the face. The camera lens was placed perpendicular to the floor, at a 90° angle [29, 30]. The participants sat comfortably on a chair, feet flat on the floor, and heads straight, with the Frankfurt plane parallel to the ground [29, 30]. A checkered banner was placed behind the participants, setting a fixed point for capturing images—their shoulders were kept parallel to the horizontal lines of the banner [24].

Participants underwent five provocative tasks—two of sustained contraction and three of functional performance (chewing)—to capture images. The sustained contraction tasks were based on the surface electromyography protocol [31], and the chewing tasks were based on the functional surface electromyography protocols [32, 33]. All participants followed the same order for obtaining the images, as follows: lip compression, lip protrusion, chewing peanuts, chewing sandwich cookies and chewing a bread roll.

The literature suggests three contractions in sustained contraction tasks, followed by a rest to obtain the desired information [31], and a portion of each food item in chewing tasks [32, 33]. Moreover, blood flow increases after muscle contraction, with a delay before temperature rises. Hence, it was decided to double the provocative tasks, believing that the temperature change would be more easily perceived with this number of contractions [6, 13, 14, 15].

Participants completed the medical history survey during acclimatisation to gather personal and health data and were positioned in front of the thermographic camera, receiving instructions on the provocative task procedure.

For the first task (lip compression), participants were instructed to sustain the contraction for 5 s and rest for 10 s before the next contraction. The images were taken immediately after each contraction, except for the pre‐test image, as follows: the first image (labelled P1.1) was obtained before the first contraction, at time zero (T0); first 5‐s contraction; the second image (P1.2) was taken, followed by a 10‐s rest; second 5‐s contraction; the third image (P1.3) was obtained, followed by a 10‐s rest; third 5‐s contraction; the fourth image (P1.4) was obtained, followed by a 10‐s rest; fourth 5‐s contraction; the fifth image (P1.5) was obtained, followed by a 10‐s rest; fifth 5‐s contraction; the sixth image (P1.6) was obtained, followed by a 10‐s rest; sixth 5‐s contraction; the seventh image (P1.7) was obtained, followed by a 10‐s rest—totalling six contractions followed by six rests, with seven images taken. There was a 2‐min break for the orbicularis oris muscle to rest.

After the 2‐min break, they performed the second sustained contraction task (lip protrusion), following the same sequence of contractions, rests and image capture. The images in the second task were labelled as follows: P2.1, before the first contraction, at time zero (T0); P2.2, after the first contraction; P2.3, after the second contraction; P2.4, after the third contraction; P2.5, after the fourth contraction; P2.6, after the fifth contraction; and P2.7, after the sixth contraction. Thus, as in the first provocative task, six contractions were performed, followed by six rests, with seven images taken.

Next, after another 2‐min break, the chewing functional tasks were performed with three different foods: peanuts, sandwich cookies, and a bread roll—peeled, roasted, and unsalted peanuts [32, 34]; Bono brand chocolate‐cream cookies [32]; and 15‐g portions of bread rolls [34]. The 2‐min interval for muscle recovery was likewise respected in the transition between all foods, as recommended in surface electromyography tests [31, 34]. The researcher purchased bread from the same bakery on their way to the collection laboratory, and it was always served fresh.

In the first functional task (peanut chewing), an image (P3.1) was obtained at rest, before the first peanut serving (T0). Then, the participant was instructed to chew a portion of peanuts, served on a dessert spoon. After swallowing this portion, another image was obtained (P3.2). There was a 30‐s interval before serving the second portion of peanuts, obtaining the third image (P3.3) after this chewing. Thus, three images were obtained for peanut chewing: one before starting the task, one after chewing the first portion of peanuts, and the third after chewing the second portion over time.

Three thermographic images were also obtained in the second functional task (chewing the Bono chocolate cookie): one at rest before starting (P4.1); after chewing the first cookie, the second image was obtained (P4.2), followed by another 30‐s interval for muscle rest. Then, the participant ate the second cookie, and the third image (P4.3) was taken. They were not instructed on how to eat the cookie, only that they should eat it in their usual way.

Lastly, three thermographic images were obtained in the third functional task (chewing bread)—one at rest, before starting (P5.1); one immediately after chewing the first bread portion and before the 30‐s rest (P5.2); and the last thermographic image after chewing the second bread portion (P5.3). As with the cookies, no specific instructions were given on how to bite and chew the bread—they were instructed to eat it in their usual way.

The thermographic images were saved in JPEG (Joint Photographic Experts Group) format in individual files per participant and exported to the Visionfy program (Thermofy, Brazil).

The orbicularis oris muscle was analysed in anatomical thermal areas, as recommended by Salles et al. [24], using the breast 1 colour scale. A total of 1288 thermograms were evaluated (23 thermograms from each of the 56 participants), following the sequence of provocative tests described above.

**FIGURE 1:**
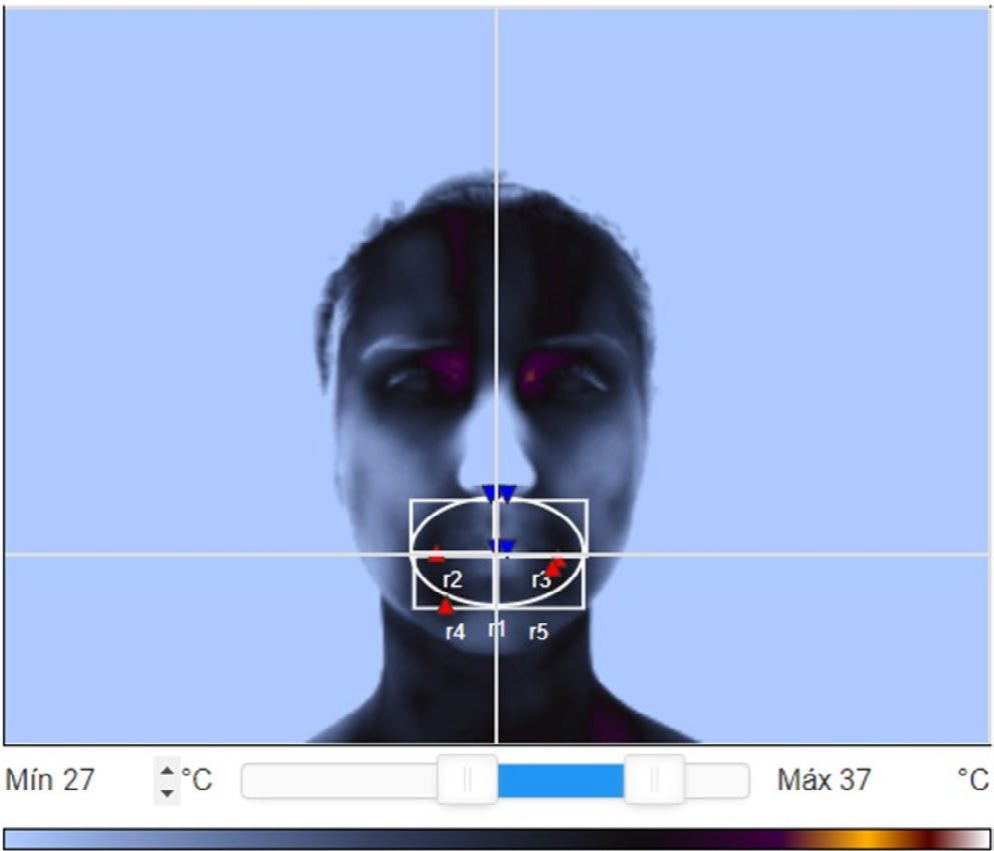
Description of the regions of interest analysed. R2—upper right rectangle, R3—upper left rectangle, R4—lower right rectangle; R5—lower left rectangle.

The following anatomical thermal areas corresponding to the regions of interest (ROIs) were studied for data extraction: R2—upper right rectangle, R3—upper left rectangle, R4—lower right rectangle, and R5—lower left rectangle [24], as illustrated in Figure 1, always using a temperature window from 27°C to 37°C.

After defining each anatomical thermal area, the study obtained qualitative thermographic data and quantitative mean temperature data (MEAN T). As indicated by the literature, the study used mean temperatures because they have lower data variability [29, 30, 35], and raw instead of normalised temperatures, as both provide the same information [13, 23].

**FIGURE 2:**
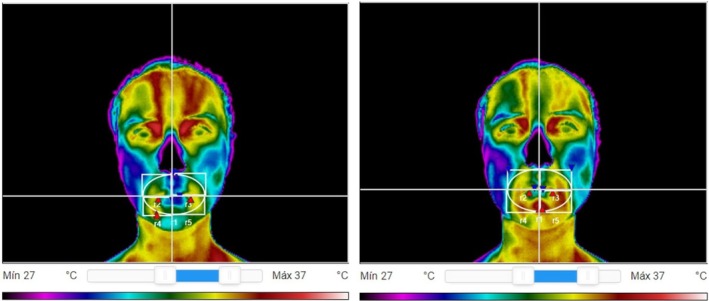
(A) First image, obtained before starting each provocative test. (B) Last image, obtained at the end of each test.

The thermograms initially underwent qualitative analysis [36], checking whether the temperature increased in the region of the orbicularis oris muscle after each provocative test (Figure 2). The qualitative analysis, conducted by a single evaluator, was best observed using the thermoguided colour scale 1, as the contrast is more easily perceived between vibrant colours than in grayscale. In this scale, black, pink, and dark blue correspond to the lowest temperatures; light blue, green, and yellow correspond to medium temperatures; and orange, red, and white correspond to the highest temperatures, as shown in Figure 2.

The temperature of each area was compared throughout the provocative tests—that is, R2 was compared between P1.1, P1.2, P1.3, P1.4, P1.5, P1.6, and P1.7 during the lip compression provocative test. It was likewise compared between P2.1, P2.2, P2.3, P2.4, P2.5, P2.6, and P2.7 during lip protrusion. This procedure was repeated in the functional tests—that is, R2 was compared between P3.1, P3.2, and P3.3 (peanut chewing), P4.1, P4.2, and P4.3 (Bono chocolate cookie chewing), and finally, P5.1, P5.2, and P5.3 (bread chewing). The mean temperatures for the other areas (R3, R4, and R5) were then compared in the same way, aiming to show the continuous change in temperature of the orbicularis oris muscle over time.

The mean temperatures of each provocative test were also compared with their respective initial values. That is, P1.1 was compared with P1.2; then P1.1 was compared with P1.3, followed by a comparison with P1.4, and so on until P1.7. The same process was repeated for all other tests and the four quadrants studied.

Moreover, the beginnings of all tests were compared with each other to verify the temperature variation between provocative tests, the accumulated temperature between tests, and whether the rest period was sufficient. That is, P1.1 was compared with P2.1; P2.1 was compared with P3.1; P3.1 was compared with P4.1; and finally, P4.1 was compared with P5.1 in each quadrant.

Two researchers with over 4 years of experience in thermography independently obtained 20% of the quantitative data to assess intrarater and interrater agreement [14] using the intraclass correlation coefficient (ICC), considering the following limits: 0–0.5 Poor; 0.5–0.75 Moderate; 0.75–0.9 Good; and ≥ 0.9 Excellent [37]. Both intrarater and interrater agreement was considered excellent—that is, the ICC was ≥ 0.9 for mean temperatures.

Next, the study performed the Shapiro–Wilk test and evaluated the histograms to verify the normality of the data. Following all guidelines for choosing statistical tests, the Student's *t*‐test was selected to compare the dependent samples—temperature variation throughout each provocative test. All tests were performed using Stata 16.0.

## 3 Results

The qualitative analysis found a change in the calorimetric scale of the orbicularis oris muscle region throughout all provocative tests. All of them (100%), taken individually, had an increase in local temperature.

**FIGURE 3:**
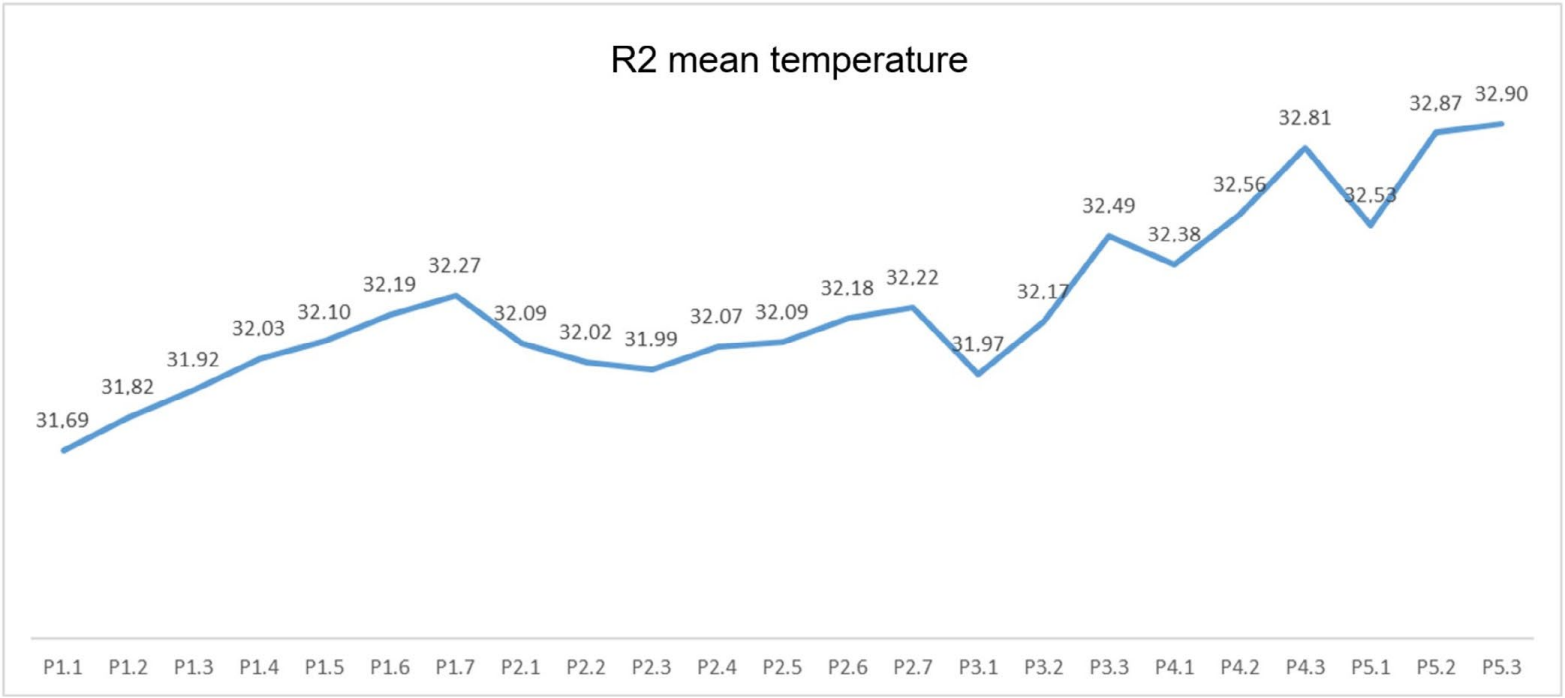
Graph of the variation in mean R2 temperatures over the five provocative tests.

In the quantitative analysis, the averaged mean temperature from the beginning to the end of each provocative test was 0.57°C in lip compression, 0.20°C in lip protrusion, 0.57°C in peanut chewing, 0.43°C in cookie chewing, and 0.37°C in bread chewing. The mean temperature analysis per anatomical thermal area identified a continuous increase in temperature throughout the provocative tests, except for the second test (lip protrusion), whose temperature decreased slightly before increasing again (Figure 3). Figure 3 shows the mean temperature variation in R2, whose pattern was similar in R3, R4, and R5. Despite the decrease in temperature in the second provocative test, the difference between all temperatures in each test analyzed individually was significant (*p*‐value < 0.05). This indicates that the tests caused a real change in temperature.

The sequential comparison (i.e., over time) of the mean temperatures of each anatomical thermal area (Table 1) shows significant variations throughout almost all tests. No difference was observed between the mean R2 temperatures in the comparison between P2.2 and P2.3, P2.4 and P2.5, and P5.2 and P5.3. There was also no difference between P2.6 and P2.7 and P3.3 and P4.1, although they were close to the established cutoff. Furthermore, there was no significance in the analysis of R3 comparing P1.7 with P2.1, P2.2 with P2.3, P2.4 with P2.5, P3.3 with P4.1, and P5.2 with P5.3. Regarding R4, no significance was found between P2.1 and P2.2, P2.2 and P2.3, and P5.2 and P5.3. Finally, regarding R5, no significance was found between P1.7 and P2.1, P2.1 and P2.2, P2.2 and P2.3, and P5.2 and P5.3.

**TABLE 1:** Sequential comparison between mean temperatures per anatomical thermal area.

Area	R2			R3			R4			R5		
	Mean	Seq diff	*p*	Mean	Seq diff	*p*	Mean	Seq diff	*p*	Mean	Seq diff	*p*
P1.1	31.69			31.83			32.19			32.33		
P1.2	31.82	0.123	< 0.001	32.01	0.179	< 0.001	32.28	0.084	< 0.001	32.46	0.132	< 0.001
P1.3	31.92	0.101	< 0.001	32.09	0.081	< 0.001	32.41	0.132	< 0.001	32.55	0.092	< 0.001
P1.4	32.03	0.117	< 0.001	32.19	0.098	< 0.001	32.50	0.089	< 0.001	32.66	0.107	< 0.001
P1.5	32.10	0.063	0.016	32.26	0.073	< 0.001	32.59	0.092	< 0.001	32.74	0.078	< 0.001
P1.6	32.19	0.099	< 0.001	32.36	0.102	< 0.001	32.68	0.091	< 0.001	32.82	0.087	< 0.001
P1.7	32.27	0.071	0.013	32.42	0.058	0.015	32.78	0.094	< 0.001	32.88	0.055	0.014
P2.1	32.09	−0.178	0.007	32.32	−0.096	0.093	32.56	−0.220	0.002	32.79	−0.090	0.103
P2.2	32.02	−0.070	0.012	32.26	−0.059	0.005	32.52	−0.033	0.131	32.76	−0.025	0.174
P2.3	31.99	−0.027	0.371	32.23	−0.038	0.106	32.54	0.016	0.466	32.78	0.014	0.493
P2.4	32.07	0.082	0.004	32.30	0.074	0.001	32.61	0.071	0.002	32.85	0.074	0.000
P2.5	32.09	0.018	0.447	32.33	0.030	0.147	32.69	0.078	< 0.001	32.92	0.070	0.001
P2.6	32.18	0.088	< 0.001	32.43	0.103	< 0.001	32.75	0.067	0.010	32.99	0.062	0.010
P2.7	32.22	0.042	0.059	32.48	0.050	0.025	32.83	0.076	< 0.001	33.05	0.060	0.002
P3.1	31.97	−0.250	< 0.001	32.20	−0.288	< 0.001	32.42	−0.406	< 0.001	32.69	−0.360	< 0.001
P3.2	32.17	0.197	< 0.001	32.40	0.204	< 0.001	32.73	0.309	< 0.001	32.95	0.266	< 0.001
P3.3	32.49	0.318	< 0.001	32.67	0.268	< 0.001	33.11	0.379	< 0.001	33.30	0.352	< 0.001
P4.1	32.38	−0.107	0.058	32.58	−0.085	0.089	32.95	−0.166	0.006	33.20	−0.102	0.048
P4.2	32.56	0.184	< 0.001	32.73	0.149	0.001	33.17	0.222	< 0.001	33.42	0.216	< 0.001
P4.3	32.81	0.247	< 0.001	32.97	0.239	< 0.001	33.42	0.251	< 0.001	33.64	0.217	< 0.001
P5.1	32.53	−0.283	< 0.001	32.73	−0.240	< 0.001	33.10	−0.316	< 0.001	33.38	−0.259	< 0.001
P5.2	32.87	0.339	< 0.001	33.04	0.305	< 0.001	33.47	0.369	< 0.001	33.73	0.352	< 0.001
P5.3	32.90	0.034	0.514	33.06	0.023	0.618	33.52	0.043	0.442	33.76	0.027	0.533

*Note:* R2—upper right rectangle, R3—upper left rectangle, R4—lower right rectangle; R5—lower left rectangle; Seq diff—Sequential difference; P1.1—Initial rest; P1—lip compression test; P2—lip protrusion test; P3—peanut chewing test; P4—cookie chewing test; P5—bread chewing test; 1 to 7—sequence of contractions; 2 and 3—food servings for chewing; Student's statistical *t*‐test, value < 0.05.

The comparison between each test and its respective resting value (T0) found that all *p*‐values were significant, showing a difference between the resting temperature and the temperature obtained after each test (Table 2). However, the P2.1, P3.1, P4.1, and P5.1 temperatures did not return to the resting temperature that preceded all provocative tests.

**TABLE 2:** Comparison of mean temperatures per anatomical thermal area between the initial values of each test and the initial value of all of them.

Area	R2		R3		R4		R5	
	In T0 diff	*p*	In T0 diff	*p*	In T0 diff	*p*	In T0 diff	*p*
P1.1								
P2.1	0.40	< 0.001	0.50	< 0.001	0.36	< 0.001	0.46	< 0.001
P3.1	0.28	0.011	0.37	< 0.001	0.23	0.0033	0.36	< 0.001
P4.1	0.69	< 0.001	0.75	< 0.001	0.75	< 0.001	0.87	< 0.001
P5.1	0.84	< 0.001	0.90	< 0.001	0.91	< 0.001	1.05	< 0.001

*Note:* R2—upper right rectangle, R3—upper left rectangle, R4—lower right rectangle; R5—lower left rectangle; In T0 diff—difference between the initial value of the tests and the initial values of each provocative test separately; P1.1—initial rest; P2.1—rest before lip protrusion; P3.1—rest before peanut chewing; P4.1—rest before cookie chewing; P5.1—rest before bread chewing; Student's statistical *t*‐test, value < 0.05.

## 4 Discussion

The qualitative analysis of thermograms allows the researcher to visualise the temperature variation through changes in the calorimetric scale [15, 36]. This research visually verified such variation on this scale, as the orbicularis oris muscle became more hyper‐radiant with each provocative test, shifting from shades of green and yellow to shades of red and even white. The qualitative analysis also supports the quantitative data showing an increase in temperature. Hence, patients can see the result of their exercise through changes in thermogram colours, providing an interesting biofeedback tool that may help improve treatment adherence. Treatment adherence is known to increase when patients understand what they are doing and what is happening in their body [38].

The orbicularis oris muscle is vascularized by the superior and inferior labial arteries, both of which derive from the facial artery, which, in turn, branches off from the external carotid artery [23, 39, 40]. Since they are part of the same arterial supply, temperatures are expected to increase and decrease simultaneously in the upper and lower lips and the right and left sides, characterising thermal symmetry [6, 15, 16, 27]. This research identified the same temperature change pattern in the four quadrants studied with the provocative tests and their respective resting phases, supporting the literature on harmonic temperature changes due to the contraction of muscles supplied by the same arterial tree.

Exercise physiology describes responses to exercise and muscle training and explains their mechanisms [41]. Muscle contractions can be isometric or isotonic, depending on the expected final result [42]. Additionally, strength training is influenced by both mechanical and metabolic stimuli. Mechanical stimulus is directly influenced by the amount of weight and number of repetitions, while metabolic changes play an important role in strength gain [43, 44, 45]. Depending on the type of exercise and its intensity, the skin temperature in the area adjacent to the muscle involved in the exercise may decrease or increase due to the combination of factors such as metabolism, muscle contraction, sweat, and skin blood flow [46].

The individual recruits a minimal number of motor units at rest, altered by isolated muscle contraction or during function [9]. In both cases, there is an increase in blood flow due to muscle action, increasing local temperature [6, 13, 14, 15]. The first provocative test (lip compression) is a type of sustained contraction, understood as an isometric contraction [42]. Thus, this first provocative test led to a constant increase in temperature, corroborating the literature that states that muscle contraction increases blood flow and, consequently, the local temperature. However, the analysis of the second provocative test (lip protrusion) found an initial drop in temperature before it began rising again. No data were found in the literature to explain this occurrence. Hence, some hypotheses were raised to try and explain this event: possible muscle fatigue, the need for the second sustained contraction test to be much more intense than the first, or an insufficient rest interval.

The functional chewing tests were then conducted in the same line of reasoning (regarding the increased temperature due to increased blood flow in relation to the work required by the muscle) [6, 13, 14, 15]. Hence, greater temperature changes were expected because this function demands more from the orbicularis oris muscle. The increase or decrease in blood circulation leads to different metabolic responses in the muscle for strength training [44]. As they chewed the three types of solid foods, the temperature increased gradually as expected, due to the different stimuli and multiple contractions exerted by the orbicularis oris, corroborating the literature. However, the local temperature increased less during the second portion of bread. It is believed that at this point, after intensive use of the orbicularis oris muscle, the temperature reached a plateau, as there is clearly a less intense upward curve in temperature compared to the previous tests.

The sequential analysis of temperature increase found that temperature differences were significant in almost all tests, in all areas. It is believed that, except for the second portion of bread, the lack of significance in the other few measures occurred by chance, as there was no defined pattern between the tests and areas. The comparison between tests and their resting values found that all data were significant—that is, each muscle action was already different from the resting state. These data confirm the previous finding regarding the increase in temperature because if there was a statistically significant difference between one contraction and another, the accumulated actions would also be significant. This finding allows clinical speech‐language‐hearing pathologists to determine the level of difficulty of the muscle action to be employed with the orbicularis oris muscle. The effectiveness of any training program involves the correct application of scientific principles in its organisation, which depends on proper control of variables such as intensity, volume, recovery interval, and training frequency [46, 47].

Finally, the comparison of all resting moments, whether initial (before any test) or relative (before each test), found significance between all values. That is, each relative rest was not the same as the initial rest, confirming the accumulation of temperature between tests. Therefore, it is believed that the muscle rest interval between tasks was not enough for the muscle temperature to return to its basal state [5, 46]. There was a greater drop in temperature before beginning the first chewing test due to the longer rest period between tests.

There is still a lack of greater technical and scientific support regarding the sequence, type of contraction, knowledge of signs and symptoms of muscle fatigue, and tone exercise by speech‐language‐hearing pathologists working with oral motor therapy. There is also a need for greater knowledge about exercise physiology in this area [41]. Thus, this research aimed to initiate knowledge about the thermographic behaviour of the orbicularis oris muscle, seeking to understand the irrigation factors that may change muscle behaviour in response to adequate functional training.

Two researchers with more than 4 years of experience with thermographic studies independently analyzed 20% of the sample [14]. The fact that they found an excellent level of intrarater and interrater agreement shows that this analysis method is reproducible [37].

As limitations of this study, we can cite the sample with only young women and the same order of presentation of the provocative tests. It is suggested that future research include other age groups and males and present the provocative tests randomly to identify temperature accumulation patterns and define the rest time. The qualitative analysis performed by only one researcher, not constituting a blinded analysis, can also be considered a limitation of the study. It is also necessary to make some adjustments in the analysis with other factors that may influence skin temperature, such as body fat percentage, the time when images are captured (due to the influence of the circadian cycle), and the stage of the participant's menstrual cycle [5].

## 5 Conclusion

This study led to the conclusion that the orbicularis oris muscle temperature increased with the provocative sustained contraction tests during lip compression and chewing. In the lip protrusion test, the temperature initially decreased before increasing. The 2‐min interval between tests was not enough for the resting temperature to return to the baseline temperature. It was found that thermography effectively identifies these temperature changes, both qualitatively and quantitatively.

## Author Contributions

The authors Patrícia Vieira Salles, Amanda Freitas Valentim and Maria Luiza Neves Caldeira declare that they are responsible for preparing the manuscript entitled ‘Thermographic behaviour of the orbicularis oris muscle under different provocative tests’, being responsible for the collection, analysis, interpretation of data and writing. Denise Sabbagh Haddad, Renata Maria Moreira Moraes Furlan, Andréa Rodrigues Motta were the project supervisors and critically reviewed the article.

## Conflicts of Interest

The authors declare no conflicts of interest.

## Data Availability

The data that support the findings of this study are available on request from the corresponding author. The data are not publicly available due to privacy or ethical restrictions.
